# Characterisation and Validation of Insertions and Deletions in 173 Patient Exomes

**DOI:** 10.1371/journal.pone.0051292

**Published:** 2012-12-14

**Authors:** Francesco Lescai, Silvia Bonfiglio, Chiara Bacchelli, Estelle Chanudet, Aoife Waters, Sanjay M. Sisodiya, Dalia Kasperavičiūtė, Julie Williams, Denise Harold, John Hardy, Robert Kleta, Sebahattin Cirak, Richard Williams, John C. Achermann, John Anderson, David Kelsell, Tom Vulliamy, Henry Houlden, Nicholas Wood, Una Sheerin, Gian Paolo Tonini, Donna Mackay, Khalid Hussain, Jane Sowden, Veronica Kinsler, Justyna Osinska, Tony Brooks, Mike Hubank, Philip Beales, Elia Stupka

**Affiliations:** 1 UCL Genomics, University College London, London, United Kingdom; 2 Division of Research Strategy, University College London, London, United Kingdom; 3 GOSgene, UCL Institute of Child Health, University College London, London, United Kingdom; 4 Centre for Translational Genomics and Bioinformatics, San Raffaele Scientific Institute, Milan, Italy; 5 UCL Institute of Child Health, University College London, London, United Kingdom; 6 UCL Institute of Neurology, University College London, London, United Kingdom; 7 Department of Psychological Medicine, Cardiff University, Cardiff, United Kingdom; 8 Blizard Institute of Cell and Molecular Science, Barts and The London, London, United Kingdom; 9 Translational Oncopathology, National Cancer Research Institute (IST), Genova, Italy; 10 Institute of Ophthalmology, University College London, London, United Kingdom; 11 Cancer Institute, University College London, London, United Kingdom; Wellcome Trust Sanger Institute, United Kingdom

## Abstract

Recent advances in genomics technologies have spurred unprecedented efforts in genome and exome re-sequencing aiming to unravel the genetic component of rare and complex disorders. While in rare disorders this allowed the identification of novel causal genes, the missing heritability paradox in complex diseases remains so far elusive. Despite rapid advances of next-generation sequencing, both the technology and the analysis of the data it produces are in its infancy. At present there is abundant knowledge pertaining to the role of rare single nucleotide variants (SNVs) in rare disorders and of common SNVs in common disorders. Although the 1,000 genome project has clearly highlighted the prevalence of rare variants and more complex variants (e.g. insertions, deletions), their role in disease is as yet far from elucidated.

We set out to analyse the properties of sequence variants identified in a comprehensive collection of exome re-sequencing studies performed on samples from patients affected by a broad range of complex and rare diseases (N = 173). Given the known potential for Loss of Function (LoF) variants to be false positive, we performed an extensive validation of the common, rare and private LoF variants identified, which indicated that most of the private and rare variants identified were indeed true, while common novel variants had a significantly higher false positive rate. Our results indicated a strong enrichment of very low-frequency insertion/deletion variants, so far under-investigated, which might be difficult to capture with low coverage and imputation approaches and for which most of study designs would be under-powered. These insertions and deletions might play a significant role in disease genetics, contributing specifically to the underlining rare and private variation predicted to be discovered through next generation sequencing.

## Introduction

The progressively decreasing costs of next generation sequencing, combined with targeted approaches such as exome sequencing, have allowed rapid deployment of this technology in a variety of contexts: population studies, familial cases of disease, as well as complex diseases. Exome sequencing represents a cost-effective strategy for identification of causal variants, especially in a clinical context [1] [2], where clinical information and familial history may aid in the identification of the causal genetic variant within a coding region.

Several population-based studies have so far provided a general overview of variation in the human genome. The 1,000 Genomes Consortium has provided the first whole genome overview in control populations indicating that the majority of SNVs are already found in dbSNP (87.28%) [3]. Similar results were reported in smaller scale studies comparing 10 disease genomes and 10 control genomes [4]. Another study on exome sequencing on a control population of 200 individuals from Denmark, with an average coverage of 12× fold, showed an excess of SNPs annotated as low-frequency (2–5%) non-synonymous coding variants in a control population [5].

One of the first published exome sequencing studies focused on a rare dominantly inherited disorder, Freeman–Sheldon syndrome [6]. This study was the first of many studies to show the potential for direct identification of the causal gene of monogenic disorders by using exome sequencing. In the past two years more than 180 papers have been published addressing specific genetic conditions using exome sequencing in patients with an inherited disorder [7], [8], [9], producing a substantial amount of data to confirm that an average exome will contain 20,283 (±523) variants, 5 of which are usually nonsense and novel [7].

Despite the surge in published studies relating to familial cases, there are many issues pertaining to genome re-sequencing which remain unexplored. As far as complex disease genetics is concerned, exome sequencing is still in its infancy. Although several labs are involved in sequencing complex disease cases, results published so far have not succeeded in identifying major causal variants of high frequency in patients under investigation, but rather single rare variants affecting a minority of the patients significantly [10]. Additionally, most of the studies conducted so far on specific diseases focus on single nucleotide variants, rather than insertion/deletion variants (INDELs). This is due in part to the fact that insertion/deletion bioinformatics analysis pipelines are still being refined [11], [12], and additional efforts for their proper annotation is needed [13].

It has been shown, however, that this type of variation is clearly widespread and probably under-characterized: recent work identified 2 million such variants with relatively limited overlap with data from current 1000 Genome releases [14], [15]. The same study also estimated that 65% of the INDEL variants found in coding regions are rare, and a significant number of SNPs from existing genome-wide association studies (GWAS) are in linkage disequilibrium (LD) with the INDELs identified, suggesting that coding INDELs are likely to be responsible for a substantial amount of phenotypic diversity and disease genetics in humans [14]. Similar data have been shown by the 1000 Genomes Exon Pilot project group, which indicated that most INDELs are often found at low frequency [16].

A substantial contribution, has been made recently by MacArthur et al, who systematically reviewed 1,000 Genome loss-of-function (LoF) variants, which they expected to be particularly enriched for artefacts compared to the other polymorphisms [17]. They identified 1,147 high confidence LoF variants out of 2,807 screened, 326 of which were INDELs. Most of these variants are rare, being subject to purifying selection, and only 43% survived an accurate and aggressive filtering step. Their validation rate seems to be inversely correlated with variant frequency (as also shown by the 81% validation rate provided by the Exon Pilot project group for INDELs which are usually of low frequency [16]), and with their position in the gene (those at the 3′ end being the most enriched for false positives). Although the study does not investigate sequence variation and LoF variant validation in disease samples, it provides an indirect analysis of the potential role of these variants in complex disease. This is achieved by imputing in seven different disease datasets, the genotypes for 417 LoF SNVs and INDELs. This analysis, however, yielded no overall excess of association signal for LoF variants as compared to other coding variants (no data is provided for INDEL variants specifically) [18] suggesting a minor role for common loss-of-function variants in disease genetics.

On the basis of a wide collaboration across University College London, Cardiff University and San Raffaele Scientific Institute, in this study we present a focused investigation of INDEL variation across exomes sequenced at 47× average coverage from a wide range of clinical patient samples, from both familial and sporadic clinically verified complex disease cases, and an extensive validation of the common, rare and private INDEL variants identified. On average, within our study, a patient exome will present 82 novel INDELs. In total, 5,749 unique novel INDELs were identified in this study, most of which are very rare. Importantly, while common variants present high false positive rates in line with variant validation issues presented by other groups, almost all private variants validated, and a significant proportion of rare variants, were found to be true. Our study indicates a potential role for private and rare LoF INDELs in disease genetics.

## Results

**Figure 1:**
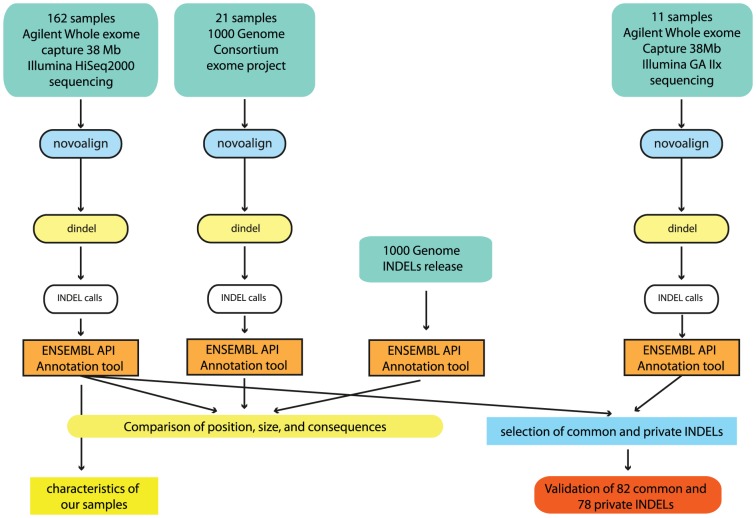
Rationale. We processed 162 exomes from different diseases (comprising 22 familial cases with mendelian inheritance and 140 sporadic/complex disease cases), and 11 samples from a different set of familial rare disorders with Mendelian inheritance, to be used for validation, with our pipeline characterised by Novoalign, Dindel and our own annotation script based on the ENSEMBL API. With the same pipeline we processed 21 samples from the Exome dataset of the 1000 Genome Consortium, to be used for comparison. 1000 Genome Consortium INDEL release October 2010 was also annotated with the same script, and annotation data have been compared. INDELs called in the two UCL datasets have been compared to identify common and private ones, and select a representative set to be validated with Sanger sequencing.

We processed 173 exomes from different diseases (comprising 33 familial cases with Mendelian inheritance and 140 sporadic/complex disease cases), using Novoalign (www.novocraft.com, previously compared on in silico data by Krawitz et al. [11]), Dindel and our own annotation script based on the ENSEMBL API (see Material and Methods). On average for each exome we obtained 36.6 millions 76 bp paired-end reads (+-2.8 millions), of which 32.1 millions mapped in proper pairs on the genome (+-2.7 millions), resulting in an average coverage of target regions of 47× (+-3.8×). With the same pipeline we also processed the Exome dataset of the 1000 Genome Consortium, to be used for our comparative analysis (Figure 1).

The dataset of disease exomes revealed an average of 20,332 SNPs which are already described in ENSEMBL, an average of 162 SNPs described only in the October 2010 release of the 1000 Genome project, and an average of 517 novel SNPs specific to our disease exome study. In total, 52,981 unique novel SNPs were identified in this study (see Text S1, section 3.2). Although our SNV data are consistent with those recently published by MacArthur and colleagues, here we focus our analysis on INDELs, thus far, largely under-investigated.

### Characteristics of INDELs

**Figure 2:**
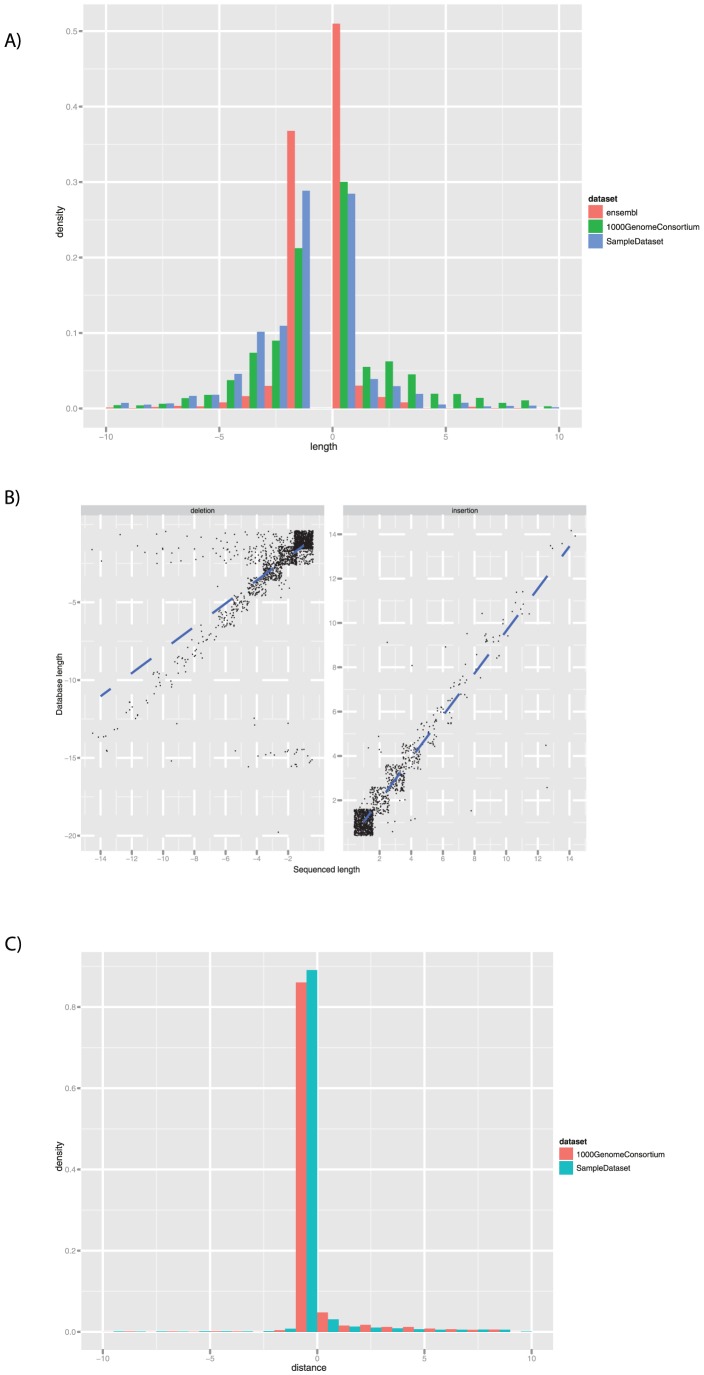
INDELs characteristics. Figure 2A shows a comparison of the length of INDEL variants called in our patients, and those available in the same capture regions in the ENSEMBL database and in the 1000 Genome Consortium release. The plot shows a higher presence of 1 bp insertion/deletions in ENSEMBL, and an increased size detection capability in 1000 Genome data, obtained from whole genome sequencing. Figure 2B shows a correlation of the INDELs already described in ENSEMBL between the size of the variant sequenced in our samples and the length reported in the database (r^2^ = 0.9221 for insertions and 0.4213 for deletions, both with p value<2* 10^−16^). Figure 2C shows the distribution of the distance (i.e. difference between start positions) between the INDELs as they were called by Dindel on our data, or as released by 1000 Genome Consortium, and the corresponding ones present in ENSEMBL.

Figure 2A in the paper shows a comparison of the density distributions of the Indel size called with Dindel on our 174-exomes sample set, those recently released by the 1000genome Consortium and those already present in the ENSEMBL database: each of these distributions takes into account the INDELs falling within our exome capture regions only. None of our calls exceeds 15 bp, and most of them have a length lower than 6 bp. Comparing our INDELS to those found in ENSEMBL it becomes apparent that ENSEMBL contains at much higher frequency INDELs of 1 bp of length as compared to the other two datasets (probably due to historical screens for single base mutations), while for all other lengths the 1,000genomes dataset includes relatively more variations than our dataset at this length: since the same caller was used (Dindel), this could be due to our choice of aligner (Novoalign), which might map less efficiently reads overlapping INDELs longer than 15 bp, as well as the use of whole genome sequencing data as opposed to exome sequencing data. This observation, coupled with the observation above that INDELs are likely to play a significant role in understanding the genetics of complex disease, indicate that improvement of sequence variation screening (by either sequencing whole genomes, improvements in read length and improvement of INDEL detection pipelines) is likely to allow a much more comprehensive characterization of the role of sequence variation in disease.

The correlation between the size of the INDEL alleles identified in our dataset and those described in ENSEMBL is high for insertions, but less so for deletions (r^2^ = 0.9221 for insertions and 0.4213 for deletions, both with p value<2* 10^−16^, Figure 2B). This highlights the difficulties the community faces in characterizing deletions appropriately, as well as the issues that it faces in using the information available in public databases to correctly characterize this type of variants. This issue is made even more challenging when taking into account that for most of the INDELs called there are multiple and different alleles, which are often not described in ENSEMBL.

Since most of the called INDELs appear to correlate with the size of those present in the database, even when multiple records are present in each window, we investigated the relationship with the distance between the start position of the INDELs called in our dataset and the closest one in ENSEMBL (Figure 2C). In order to do so, we extended the window size to 100 base pairs flanking our call and within the multiple ENSEMBL hits in our window, we selected the closest one of similar length if present and plotted its distance from the sequenced INDEL. The majority of described variations fall within 5 bp from the starting point of the INDEL called in our dataset, and most of them within 10 bp. Notably, most of the variations appear to be found only downstream of our starting point: Dindel tries to reposition each INDEL as far to the 5′ end (lower coordinates) as possible, given the same alternative haplotype (Albers C. personal communication) and left-aligning of reported INDEL position is emerging as a standard. This phenomenon is likely due to the fact that existing databases have been built much before these standards emerged and the 1,000 Genome efforts.

**Figure 3:**
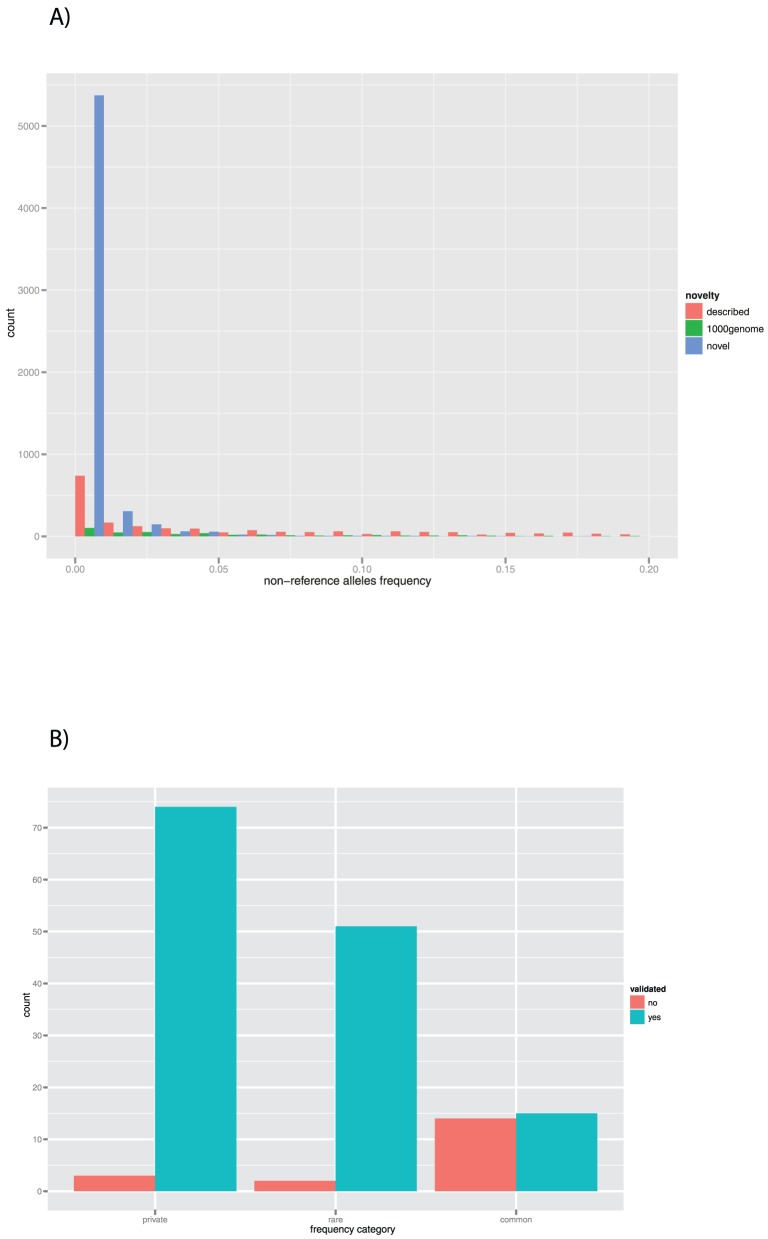
INDELs frequency and validation. Figure 3A plots the non-reference allele frequency of INDELs called in our samples, divided in three categories: those already described in ENSEMBL, those described only in the released of 1000 Genome and those completely novel to our dataset, most of which are rare. Figure 3B shows the counts of validated INDELs according to the following frequency categories: common (non reference allele frequency equal or higher than 0.05), rare (frequency lower than 0.05) and private. The validation rate is significantly different in the three groups (Chi-squared = 44.4844, p-value = 2.189*10^−10^).

We calculated the non-reference allele(s) frequency in our dataset and categorized the effect of each INDEL using the ENSEMBL API. While INDELs which are already described in existing databases such as dbSNP follow a distribution across a higher range of frequencies (Figure 3A), most of the insertions and deletions recently described by the 1,000 Genome project and those which are entirely novel to our dataset are distributed on lower frequencies (Figure 3A and Table 1, Table S1 for comparison with other datasets counts). These characteristics are in line with the most recent studies published by other groups, and in particular Mills et al [14] and MacArthur et al [18].

**Table 1:** INDELs counts.

	described in ENSEMBL			described in 1000 Genome only			novel				total per consequence	
consequence	average (std.dev)	average rare (std.dev)	Ratio rare/total	average (std.dev)	average rare (std.dev)	Ratio rare/total	average (std.dev)	average rare (std.dev)	average rare complex	Ratio rare/total	average	rare
ESSENTIAL_SPLICE_SITE	5.25 (1.43)	0.24 (0.44)	0.045714286	0.34 (0.52)	0.07 (0.26)	0.205882353	0.61 (0.84)	0.43 (0.68)	0.38 (0.65)	0.704918033	6.2	0.74
STOP	0 (0)	0 (0)		0 (0)	0 (0)		0.03 (0.21)	0.03 (0.21)	0.02 (0.19)	1	0.03	0.03
COMPLEX_INDEL	3.13 (1.01)	0.17 (0.41)	0.054313099	0.36 (0.52)	0.09 (0.29)	0.25	0.47 (0.63)	0.24 (0.43)	0.21 (0.41)	0.510638298	3.96	0.51
FRAMESHIFT_CODING	69.4 (7.38)	3.24 (2)	0.046685879	3.28 (1.73)	1.15 (1.05)	0.350609756	23.49 (5.43)	15.73 (4.89)	13.81 (5.51)	0.670940171	96.17	20.11
NON_SYNONYMOUS_CODING	68.33 (6.57)	2.59 (1.8)	0.037920937	2.24 (1.11)	0.47 (0.67)	0.209821429	6.69 (3.19)	6.08 (3.13)	5.2 (3.07)	0.908819133	77.26	9.13
SPLICE_SITE	98.42 (7.53)	3.35 (2.15)	0.034044715	4.69 (2.01)	0.97 (0.99)	0.206823028	7.05 (3)	5.21 (2.74)	4.84 (2.68)	0.739007092	110.16	9.53
5PRIME_UTR	22.78 (3.18)	0.66 (0.92)	0.02907489	0.53 (0.64)	0.14 (0.35)	0.264150943	1.37 (1.12)	1.37 (1.12)	1.28 (1.08)	1	24.69	2.17
3PRIME_UTR	49.45 (5.3)	1.32 (1.28)	0.026720648	3.08 (1.45)	0.4 (0.59)	0.12987013	2.33 (1.68)	2.18 (1.67)	2.05 (1.63)	0.935622318	54.85	3.9
INTRONIC	616.76 (32.95)	16.44 (9.63)	0.026623377	32.93 (5.55)	6.33 (2.71)	0.192401216	37.69 (11.25)	27.6 (10.81)	25.2 (10.8)	0.734042553	687.38	50.37
OTHER	12.91 (2.58)	0.6 (0.74)	0.046511628	0.36 (0.53)	0 (0)		2.02 (1.32)	0.85 (0.96)	0.78 (0.93)	0.420792079	15.29	1.45
total per category	946.42	28.61	0.030232558	47.81	9.61	0.201046025	81.76	59.72	53.77	0.730722154		

The table summarises the counts and standard deviation of INDELs according to their predicted consequence in the full dataset, in the subgroups of rare variants (i.e. MAF<0.05) and for the novel calls also the rare variants as calculated by excluding the familial cases from the dataset and re-computing the MAF. Adjusted p value indicates the results of a Wilcoxon test between rare variants in the entire dataset, and those rare in the dataset without familial cases.

### Low rate of false positives for rare and private INDELs

Taking into account data shown in other studies indicating that LoF variants may be enriched for artefacts, we proceeded to validate systematically 160 insertion/deletions in a different set of samples. By comparing the variants called in the two datasets and we were able to identify 82 INDELs which are common between the two sample sets (Table S2), and 78 INDELs which are private (Table S3). All of these variants were then validated using Sanger sequencing, both in DNA samples where they were originally called, as well as in samples where they were meant to be absent, in order to check for both false positives and false negatives. Overall the validation rate for the INDELs called using our pipeline was higher than shown in previous studies, i.e. 88.13% (Table 2). Interestingly, private INDELs presented a significantly higher validation rate (96.15%) as compared to the ones present in more than one sample (80.49%, Chi-squared = 7.9378, p-value = 0.004841). The high validation rate of private INDELs is likely due to the use of high coverage sequencing in the analysis and highlights an important portion of very rare variants that are likely to be either detected with higher false positive rates, or missed entirely by low coverage population sequencing.

**Table 2:** Validation of variants.

A) Common INDELs			
	Validated	Not Validated	Validation Rate
Novel	24	13	64.86%
Newly released	42	3	93.33%
Total	66	16	80.49%

B) Private INDELs			
	Validated	Not Validated	Validation Rate
Novel	73	3	96.05%
Newly released	2	0	100.00%
Total	75	3	96.15%
**Total**	**141**	**19**	**88.13%**

The table provides a summary of the validation results, both for the INDELs common to the two sequencing datasets used, and the private ones. All INDELs sent for validation were classified as “novel” according to dbSNP 131 and the 1000 Genome Consortium release October 2010. During the validation phase new data have been released by 1000 Genome (November 11th 2011): INDELs have been here categorised according to this latest release, to be considered as an independent confirmation.

If we analyse these data in more detail, and we take into account the frequency of the variants by dividing them into common (non-reference allele frequency equal or higher than 0.05), rare (frequency lower than 0.05) and private as shown in Figure 3B, the validation rate is significantly different between the three groups (Chi-squared = 44.4844, p-value = 2.189*10^−10^). We carried out our validation phase by genotyping the INDELs in two samples: one reported by NGS to carry the variant, and one reported as homozygous for the reference. This choice allowed us to check for false negatives. None of the samples expected to be reference reported insertion/deletions in Sanger sequencing, and was therefore confirmed as negative. In line with previous observations, most of the INDELs which did not validate displayed a high frequency in our patients, with some exceptions (i.e. a large frameshift insertion on chromosome 7 that will require further investigation (Table S2).

**Figure 4:**
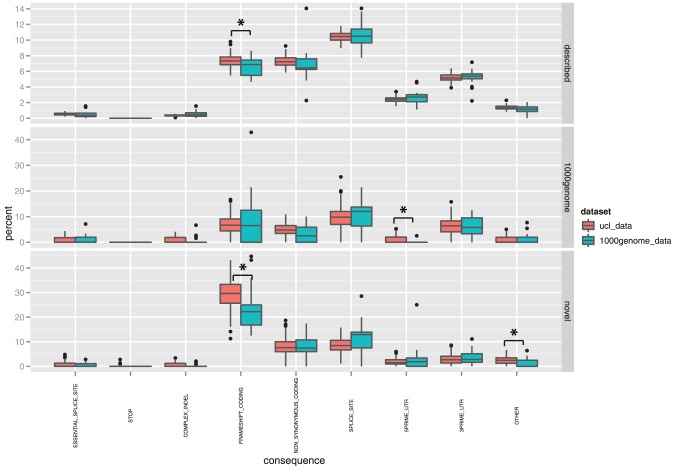
INDELs consequences comparison. This boxplot details the differences in the comparison of the distributions of consequence proportions per sample between our disease exomes data and 1000 Genome exomes. Significant differences, calculated with a non parametric Wilcoxon test of independent samples, have been highlighted with a star.

We finally performed a comparison between the distribution of the predicted consequences in our patients samples and the samples from 1000 Genomes we re-analysed: the analysis (Figure 4) highlighted a higher proportion of frameshift INDELs in our samples (Test S1, section 4.3; Table S4 and Figure S2). Although such a finding might be influenced by several differences between the two samples sets, we were unable to pinpoint any specific difference which could explain this result.

## Discussion

We present here the first overview of INDEL variations found in exomes performed at an average 47× coverage on patient samples from a variety of confirmed sporadic and familial cases of disease. The pipeline we used results in a good validation rate, as it actually yields better validation rate (88.1% against 81.3%) than previously published validation results [16]. Our analysis highlights a considerable number of variants which are not included in the 1,000 genome release. The choice of algorithms, thresholds, and filters could have an impact on our findings and the rate of false positive LoF variants identified but the results indicated the parameters adopted perform in line with other published pipelines. For this reason we decided to re-analyze the 1,000 Genome data using our pipeline, to ensure the comparison was accurate, and correct for the total number of variants called per sample. Moreover, we also tested the impact of using the GATK pipeline, which provided very similar results and did not eliminate the common false positive alleles that were identified.

In the analysis of 173 exomes it is clear that most variation identified within exome disease study as novel is of a “private” nature or of very low frequency. This confirms that the more we will sequence, the more novel alleles we will identify, as previously suggested [14]. On the other hand, despite the 1,000 Genome Project effort, we are still able to identify in our disease dataset some novel, validated, high frequency alleles. Part of this may be due to the choice of the aligner and caller combination, as our re-analysis of 1000 Genome exome shows, but on the other hand this result also indicates that obtaining and sharing more data from disease exome studies will help to have a better picture of sequence variation in disease.

While INDELs with very high frequency across different patients tend to be artifacts as indicated by our validation analysis, we noticed some high frequency INDELs which were not identified by the 1,000 G project, and might be due to differences in the bioinformatics pipeline employed. On a similar note, we identify known high frequency INDELs in our patient exomes, although they are putatively damaging. These findings highlight that the search for variants that are potentially disease-causing cannot be simplified to searching for merely “novel damaging variation”, since known variants can be causative, and damaging variants can be tolerated, as was well described recently [13], [18].

The presence of a considerable number of loss of function insertion-deletions in our samples would suggest that the genetics of complex disease are, indeed a compound effect of several rare and private more complex variants across the genome. To establish this with greater certainty, and also to understand to what extent it is a property of many diseases as opposed to specific ones, comprehensive frequency information from large control and disease populations will be of fundamental importance.

The finding that our samples are particularly enriched in validated novel and low-frequency variants is important because most of the study designs and latest statistical methods addressing the role of rare variants would still be under-powered for private or very low frequency insertion-deletions. Low-coverage and imputation methodologies would not be effective and high coverage sequencing would still be required to capture them [19], [20], [21].

Interestingly, INDELs and in particular frameshift INDELs, stand out for a variety of reasons as being frequent in our patient disease exomes. It is tempting to speculate that, if common LoF variants do not play a major role in complex disease as suggested recently [18], low frequency and, in particular, private INDELs might contribute quite significantly to as yet uncharacterized genetics of disease. In particular they might confirm the expectations from next generation sequencing to discover rare and private variants that explain and characterise individual variability in health and diseases (Figure S3), as our data suggest. Our analysis indicates, however that many challenges still exist, however, in assessing this type of variation accurately, given the current lack of frequency information in large control and disease populations, as well as their variability in length, multiple potential alleles, and position which often makes it difficult to assign a highly confident identity to an INDEL variant.

The need for appropriate control populations is highlighted by our comparison with 1,000 Genome samples. The differences we found, although interesting, might be affected by several biases difficult to identify and address despite the use of the same analysis pipeline: differences in capture technology and read length, in coverage of the coding regions, the ethnicity of the samples and mixed ancestry or other unknown technical bias. Such biases are difficult to remove, despite our careful corrections.

Importantly we were only able to identify relatively short INDELs, while the identification of much larger INDELs which are relatively common is highly affected by the choice of existing bioinformatics pipelines. Thus, our disease exome study provides a pointer to what might be the most interesting source of sequence variation in disease, insertions and deletions, and at the same time highlights many of the limits that we currently face in fully assessing this type of sequence variation. If, despite our limitations, this type of sequence variation emerges as a significant component, we can only be brought to imagine that if we had more comprehensive data (e.g. full genome sequences with longer sequence read lengths), we would see this component play an even more significant role.

On average we identified 15 rare frameshift INDELs in each patient, suggesting an important role for these variations. Our group of patients comes from different origins and certainly ethnicity may influence the discovery of novel variants, or change their frequency, and these issues should be taken into account when expanding and characterising internal collections.

Clearly there are some substantial reproducible artefacts using current sequencing technologies and bioinformatics pipelines, since certain common, high quality false positive variants recur in a large number of samples. The use of filters and automatic procedures certainly aids in reducing the observed enrichment of false positives in LOF calls [17] together with other filters recently suggested (location in the transcript, gene etc.) [18], but still does not bring validation rate to acceptable standards (e.g. greater than 95%). Interestingly, however, our study indicates that this is not the case for rare and private INDEL variants, which have remarkably high validation rate.

Once we excluded technical artefacts, the identification of novel, rare or private, potentially damaging variants across such a diverse group of diseases opens additional areas of investigation: despite their predicted effect those variants may not be causative. The observation that the consequences of novel INDELs have a higher variability across these disease patients seems to reinforce their role in this scenario. Integration of larger whole genome and exome datasets from both patients and controls will provide more clues with regards to the relationship between these variants across the genome and their link with disease.

Interestingly, although our sample collection contains samples from both sporadic cases of disease (140) and familial cases of disease (22), all of the results shown, in terms of allele consequences and frequencies are similar in both datasets. Removing the familial cases from the dataset had no significant impact on the frequencies observed for neither SNPs nor INDELs across biological consequences, dataset categories, or MAF categories. This raises some interesting questions, since so far most publications focused on rare familial disorders and thus the discovery of rare, damaging causative SNPs was expected. Our study, however, indicates that rare and potentially damaging INDEL variation is a common feature also in exomes from sporadic cases of complex disease and that, in particular, frameshift coding insertions and deletions are a specific and significant feature also in sporadic cases of complex disease.

## Materials and Methods

### Samples

The samples used in this analysis come from several different research projects: neurological disorders (Alzheimer's, Parkinson's disease, epilepsy, ataxia), muscular dystrophy, retinal dystrophy, liver cirrhosis, eczema and erythrokeratoderma.

### Sample preparation

Samples for the 162 exome dataset were sequenced using the HiSeq 2000, following Illumina supplied protocols, with 100 bp paired end kits.

Samples for the 11 exomes validation dataset were prepared by GOSgene at the UCL Institute of Child Health and sequenced using the Genome Analyzer IIx (UCL Genomics facility, at the University College London), following Illumina supplied protocols, with 76 bp paired end kits.

The sequencing data of the patients dataset was produced at the Beijing Genomics Institute and analyzed by the UCL Genomics.The sequencing data used as a validation dataset was produced and analyzed by the UCL Genomics facility, at University College London and GOSgene.

### Quality control

After the sequencing reactions were complete, the Illumina analysis pipeline was used to process the raw sequencing data (Bustard and Gerald) and produce fastq format files. The quality of the sequencing runs were assessed by evaluating the percentage of clusters passing the filter, and by running the FastQC software and evaluating read length and base quality profiles, GC content, average GC content per base, average base content per read position, and checking for any indication of over-represented sequences.

### Alignment

Once the raw sequence data was assessed for quality, the reads were aligned to a human reference genome (GRC37 release, downloaded from the ENSEMBL database). Novoalign performed gapped alignments and was launched with the additional hard clipping option based on read base quality (-H) and the default adaptor removal option (-a).

### Coverage and alignment summary

The alignment summary is reported by using in-house perl scripts, that count the bitwise flags for the sam files produced during the alignment steps.

The coverage along the genome has been calculated using BEDtools (GenomeCoverageBed function), without omitting zero values.

In-house perl scripts have been used to summarize these data in mean, median, standard deviation and percentiles across the captured regions.

### Indel calling with Dindel

Dindel version 1.01 has been used to call INDELs from Novoalign alignments. The default parameters have been used. Dindel requires a BAM file containing the read-alignments as input. It then extracts candidate INDELs from the BAM file, and realigns the reads to candidate haplotypes consisting of these candidate INDELs in windows of ∼120 bp. If there is sufficient evidence for an alternative haplotype to the reference, it will call an INDEL and produces a VCF file.

### Annotation

The annotation of both SNPs and Indel variants has been performed with an in-house perl script that integrates with the ENSEMBL API and queries the database on all available features, formats them in summary files, compares the calls with the 1000genome calls and classifies the variants (algorithm in Figure S1).

A more detailed explanation can be found in Text S1, section 5.2.

### Comparisons and reporting

The visualization and comparison of the different length and frequency distributions, as well as the predicted effect and calls have been done using R (www.r-project.org) and the graphical package “ggplot2”.

### Statistical analysis

In order to compare the proportion of different consequences across our disease dataset and the 1000 Genome exome dataset, and account for differences in the total number of variants called in each individual, we converted the counts in percentage per individual, and compared the distributions between the individuals of the two groups by using a non parametric Wilcoxon test of independent samples. The tests have been performed using R, basic “stats” package.

### Validation of the INDELs

5 ng of each DNA sample were amplified in a 20 µl reaction mixture containing 0.2 mM of each dNTP (Takara), 1× Titanium *Taq* PCR Buffer (Clontech), 0.25× Titanium *Taq* DNA Polymerase (Clontech) and 0.2 µM of forward and reverse primers designed to specifically amplify each DNA fragment containing a putative indel. The PCR program included an initial step of denaturation at 95°C for 1 minute followed by 35 cycles of amplification characterized by the following profile: 95°C for 30″, 60°C for 30″, 68°C for 30″ and a final extension step at 68°C for 3 minutes. Each PCR product was purified with Agencourt® AMPure® XP (Beckman Coulter) magnetic beads and subsequently sequenced by standard dideoxy-sequencing on Applied Biosystem 3730 with both forward and reverse primers. Sequence data obtained from each PCR fragment were aligned to the corresponding reference sequences with the software Sequencher 4.9 (Gene Codes).

## Supporting Information

Figure S1 **INDELs annotation.** The figure shows the decision process adopted in our annotation algorithm, in determining if an INDEL has been previously described in ENSEMBL or in the 1000 Genome release. Priority is given to variants of the same length, and sequence, followed by the closest variant, if present within a 10 bp window from the start position of the sequenced variant.
(EPS)

Figure S2 **INDEL consequences comparison.** In this picture, the proportion of different consequences is represented. In the first column, the INDELs available in ENSEMBL and within our capture regions are reported. The second column reports the same annotation performed on the variants released by 1000 Genome Consortium. The other groups of columns compare the consequence proportions of our analysis on 1000 Genome exome data and our patients' exomes.
(EPS)

Figure S3 **Consequences: sample level variability.** The figure shows an overview per sample of the proportion of different consequences for SNPs (A) and INDELs (B).
(EPS)

Table S1 **INDELs counts.** The table summarises the average counts (and standard deviation) of each consequence per category of dataset. Our calls have been divided among those already described in ENSEMBL, those described only in the latest release of 1000 Genome and those that are novel. The table reports the counts for the annotation of the variants available in ENSEMBL and positioned in our capture regions, the same annotation for the variants released by 1000 Genome and the comparison between our analysis of 1000 Genome data and our dataset.
(XLSX)

Table S2 **Common INDELs validation.** The table lists the INDELs common between several samples, with higher frequency in our dataset, which have been validated in the 11 samples used for validation with Sanger sequencing. The column “nonRef_MAF” reports the frequency of non-reference alleles in our samples, the column “sequence” indicates whether Sanger sequence reported the same sequence as called in NGS data, and the column “latest1000G” indicates if the variant has been called in the release of November 2011 of the 1000 Genome Consortium.
(XLSX)

Table S3 **Private INDELs validation.** The table lists the INDELs private to single samples, which have been validated in the 11 samples used for validation with Sanger sequencing. The column “nonRef_MAF” reports the frequency of non-reference alleles in our samples, the column “sequence” indicates whether Sanger sequence reported the same sequence as called in NGS data, and the column “latest1000G” indicates if the variant has been called in the release of November 2011 of the 1000 Genome Consortium.
(XLSX)

Table S4 **Comparison of INDELs consequence proportions.** The table reports the average percentage of each consequence within category of called INDELs, and within the data available in ENSEMBL and released by the 1000 Genomes Consortium. Significance values are calculated comparing the two distributions of per sample percentages, within each category, with a Wilcoxon two independent samples test.
(XLSX)

Text S1 **Supplementary analysis and description of the detailed methodology and workflow.**
(DOCX)
