# Supplementary material for: Characterisation and Validation of Insertions and Deletions in 173 Patient Exomes
Source: PLoS One. 2012 Dec 14;7(12):e51292. doi: 10.1371/journal.pone.0051292 (PMC3522676; doi:10.1371/journal.pone.0051292)
Supplement: Text S1 — Supplementary analysis and description of the detailed methodology and workflow. (DOCX) [file pone.0051292.s008.docx]

Supplementary Information

1. Data Generation 2

1.1. Disease Exomes samples 2

1.2. 1000 Genome exome data 2

2. Read Mapping 2

2.1. Reference genome 2

2.2. Aligners and aligners parameters 2

2.3. Coverage 2

3. SNPs Calling 3

3.1. Software and filtering parameters 3

3.2 SNPs characteristics 3

4. INDELs Calling 4

4.1. Software and filtering parameters 4

4.2. Comparison with 1000 Genome exomes 4

4.3. More LoF INDEL variants in disease exomes as compared to 1,000 genome project exome data 4

5. Variant annotation 5

5.1. ENSEMBL database version 5

5.2. Annotation script characteristics 5

5.3. INDELs annotation 6

6. Sample variability 7

# Data Generation

## Disease Exomes samples

The 162 samples used for the large scale analysis come from a range of different research groups, and include several disease of various nature: neurological disorders (Alzheimer’s, Parkinson’s disease, epilepsy, ataxia), muscular dystrophy, retinal dystrophy, liver cirrhosis, eczema and erythrokeratoderma. 140 of these patients are sporadic cases of complex diseases and 22 come from families with inherited disorders.

The 11 samples used for the validation phase of this analysis have been contributed by the GOSGENE project, collecting and analysing rare pathologies with next generation sequencing.

## 1000 Genome exome data

The Exome Project is a whole exome sequencing project which is targeting the CCDS gene set in 2500 individuals: the Consortium released an exome run which is part of such a project. The capture design has been created by taking the intersecton between the capture design files used by the four production centers (BCM, BGI, BI, and WUGSC) and CCDS. The version used for this analysis was built based on the GRCh37.1 (NCBI HG19) reference sequences.

Samples from BCM/BGI centres used NimbleGen SeqCap_EZ_Exome_v2 for pull down, while WUGSC/BI used Agilent SureSelect_All_Exon_V2.

In order to match exactly the technology we used, only 21 CEU samples processed with Illumina HiSeq have been downloaded from 1000 Genome ftp. The samples used for this analysis are: NA18526, NA11893, NA12347, NA12348, NA12383, NA12546, NA12749, NA12828, NA12892, NA07037, NA11932, NA11933, NA12287, NA12775, NA12775, NA12777, NA12778, NA12878, NA12399, NA11920, NA07051.

# Read Mapping

## Reference genome

The reference genome used for this analysis is the GRCh37.1, downloaded from ENSEMBL.

## Aligners and aligners parameters

The alignment has been performed using Novoalign version 2.07.00.

Novoalign has been launched with the additional option –H (quality trimming of the reads) and –a (removal of adaptors).

## Coverage

The 162 patients sample set has yielded on average 56,207,980 reads, 47,776,783 of which mapped (85%) with Novoalign, for an average coverage on the capture region, after duplicates removal of 47X.

The 11 samples from GOSgene used for validation yielded an average number of 80,694,156 reads, 73,138,377 of which mapped (91%), with an average coverage of 68X (after duplicates removal).

# SNPs Calling

## Software and filtering parameters

Samtools version 0.1.7-6 (r530) has been used for .bam files manipulation and for SNP calling from the alignments generated with Novoalign.

The Samtools internal function (rmdup) has been used in order to remove potential PCR duplicates. The new bam is then used as an input to extract a pileup with a Maq-like consensus sequence (paramenters “-vc”). Such a produced preliminary pileup output is used to filter the variant calls (function samtools.pl varFilter) with the following parameters: “-d 15 -S 30 -Q 60 -q 60”. Briefly, the parameter “-d” sets the minimum depth, “-S” the minimum SNP quality, “-Q” the minimum mapping quality for SNPs and “-q” the minimum mapping quality for gaps. The final output is then converted in VCF format.

## 3.2 SNPs characteristics

The dataset of disease exomes revealed an average of 20,332 SNPs which are already described in ENSEMBL, an average of 162 SNPs described only in the October 2010 release of the 1000 Genome project, and an average of 517 novel SNPs specific to our disease exome study. In total, 52,981 unique novel SNPs were identified in this study.

We then used the ENSEMBL API to predict the effect of the SNP calls on potential transcripts as annotated in the database. The biological consequence of the SNP is ordered in terms of its potential biological effect for loss of function. We grouped within “other” those variants with putatively minor consequences as described by ENSEMBL. We downloaded all the SNPs available in ENSEMBL (derived from dbSNP, HapMap and other sources often also containing many disease causing variants) and the 1000 Genome Consortium October 2010 release, located the variants in our exome capture regions, and annotated them in the same way.

In order to account for potential biases in comparing variants called with different methods, we downloaded and re-analysed with our pipeline high coverage exome data (data comparable to ours) released by the 1,000 Genome project. We therefore compared the consequences of the variants called in our patients dataset to those of the ENSEMBL database, those of the variants in the October 2010 release of 1000 Genome, and those of the variants called in our re-analysis of 1000 Genome exome data.

Most of the characteristics of the SNPs, in terms of distribution of the consequences across the frequency spectrum, have been previously reported in the literature, and our data can only confirm previous observations.

No major differences can be observed.

In detail, significant but minor differences have been observed mostly within SNPs already described in ENSEMBL: variations with an essential splice site consequence were 0.19% in 1000 Genome re-analysed samples and 0.17% in our patients’ samples, stop codons 0.66% against 0.7%, and non synonymous coding which displayed the highest difference were 30.1% in 1000 Genome samples against 31.45% in our collection. Other small differences have been reported in splice site (5.05% against 4.96%), synonymous coding (30.39% against 31.05%), 5’ UTRs (1.68% against 1.35%) and intronic variants (25.54% against 24.02%). The only significant difference observed in novel variations was among synonymous coding SNPs, representing 45.54% in 1000 Genome re-analysed samples and 48.17% in our samples, and intronic SNPs (21.5% against 18.9%).

# INDELs Calling

## Software and filtering parameters

Samtools version 0.1.7-6 (r530) and Dindel version 1.01 have been used to remove the duplicates and then call INDELs from the output of Novoalign.

Dindel has been launched with its default parameters.

## Comparison with 1000 Genome exomes

Figure 4A in the paper gives a visual representation of the details reported in Table S2. The p values indicated in the table have been determined by calculating per individual percentages of each consequence, and using these distribution of consequence values across all individuals of our dataset, and those of the 1000 Genome exome dataset to perform a Wilcoxon two-independent sample test. Among variants already described in ENSEMBL, frameshift INDELs are higher in our patients (p. value = 3.9*10^-2^). Among those described only in the latest 1000 Genome release, there is no significant difference between the two datasets. Among the novel variants, as described in the paper, frameshift coding INDELs are significantly higher in your samples (p. value = 2.9*10^-4^).

## More LoF INDEL variants in disease exomes as compared to 1,000 genome project exome data

In an average clinical exome, 946 of the INDELs identified are already described in ENSEMBL, 48 are described only in the 1,000 Genome data and 82 are only found in our disease exome dataset (Table 1). Altogether in our 173 samples we find 5,749 such INDELs, i.e. identified in our clinical samples, but not previously described. Since we re-analyzed 1,000 genome project exome data with our own pipeline, we also identify 654 INDELs which were previously not described in the 1000 Genome exome data, of which 381 are also found in our disease exome dataset. Non-intronic INDELs can be further categorized into frameshift and in-frame (i.e. 3n long, classified as non-synonymous coding) categories (Figure S2 and Table S1), where frameshifts are clearly more likely to disrupt the protein. While in ENSEMBL, a vast majority of INDELs are frameshift coding insertions/deletions (49.56%), in our exome data they make up only 7.3% of the INDELs described in ENSEMBL, 6.9% of those described only in the 1,000 Genome release and 28.7% of novel variants specific to our dataset (Figure S2, and Table S2). The higher rate of frameshift INDELs in disease exomes is still highly evident when we compare our dataset with the 1,000 Genome exomes re-analysed using our pipeline and corrected for the differences in total INDEL counts per sample (24.8% novel frameshift INDELs in 1,000 Genome data as compared to 28.75% in UCL disease exomes, p. value = 3.6*10^-4^, Figure S2 and Figure 4 in the paper). Thus novel frameshift INDELs are more frequent in our disease exomes than in the 1,000 genome population data.

Interestingly we observe that the distribution of the biological consequence annotated for INDELs across individual samples shows greater variability for novel variants as compared to described ones (see Figure S3B).This can be explained by the fact that most of the novel ones are also rare, and they may account for an important component of individual diversity. This observation would be in line with the suggestion made by MacArthur et al. that common LoF are likely to play minor role in complex diseases. The two studies taken together would rather suggest that individual diversity and genetic risk for disease are more likely to be the result of a combination of variants which are rare or entirely private to the individual.

Both datasets have been processed with the same pipeline: additionally the coverage of UCL exomes was 47X and 1000 Genome exomes 202X. The comparison has been performed on variants called on the same regions, i.e. the areas captured by the Agilent SureSelect 32Mb, while 1000 Genome samples cover a larger area (90% of 1000Genome consensus capture have non-zero coverage from the Agilent capture, as calculated with BedTools coverageBed – mean value).

Despite this careful analysis, it is not possible to assess the extent of bias in comparing samples processed true different laboratory protocols and sequencing pipelines, as well as different ethnicities, known to influence the absolute number of INDELs in the sample. These comparison therefore, despite intriguing, can only suggest an interest for the role of INDELs in disease and stimulate further investigations on their variability across samples and different disorders (see paragraph 6).

# Variant annotation

## ENSEMBL database version

To annotate our results we used the ENSEMBL database version homo_sapiens_core_61_37f, with the corresponding ENSEMBL api version 61.

## Annotation script characteristics

In order to annotate the results we used an in-house built perl script that queries the ENSEMBL database via the perl API developed by ENSEMBL.

The script performs a first check on whether the variant to be annotated is already present in the database, in the 1000 Genome release of October 2010, or it is completely new. A rich genome annotation is produced, including nearest gene, overlapping transcripts, population frequency data, HapMap validation etc.

A particular importance is given to the prediction of the consequence, both for known and novel variants, possible using the ENSEMBL API.

In case of multiple consequences on different transcripts, the most “damaging” is selected, according to a scale of functional priorities set by ENSEMBL.

The following consequences are annotated:

1. Essential splice site: in the first 2 or the last 2 basepairs of an intron.
2. Stop gained: in coding sequence, resulting in the gain of a stop codon (i.e. leading to a shortened peptide sequence).
3. Stop lost: in coding sequence, resulting in the loss of a stop codon (We grouped together stop consequences under the term “stop”).
4. Complex InDel: insertion or deletion that spans an exon/intron border or a coding sequence/UTR border.
5. Frameshift variations: in coding sequence, resulting in a frameshift.
6. Non-synonymous SNPs: SNPs that are located in the coding sequence and result in an amino acid changein the encoded peptide sequence.
7. Splice site: 1-3 bps into an exon or 3-8 bps into an intron.
8. Partial codon: located within the final, incomplete codon of a transcript with a shortened coding sequence where the end is unknown.
9. Synonymous SNPs: in coding sequence, not resulting in an amino acid change (i.e. silent muta-tion).
10. Regulatory region variations: in regulatory region annotated by Ensembl.
11. Within mature miRNA: located within a microRNA
12. 5prime UTR variations: in 5prime UTR (untranslated region).
13. 3prime UTR variations: in 3prime UTR.
14. Intronic variations: in intron.
15. NMD transcript: located within a transcript predicted to undergo nonsense-mediated decay.
16. Within non-coding gene: located within a gene that does not code for a protein.
17. Upstream variations: within 5 kb upstream of the 5prime end of a transcript.

Variations with very low frequency such as regulatory regions, miRNA, NMD transcript, non-coding gene and partial codon have been grouped together under the term “other” in the aggregated analysis and plots.

## INDELs annotation

Since the accurate detection of the correct length and size of INDELs can be challenging, we defined an INDEL as “described” if it was found in the ENSEMBL database within 10 base pairs on both sides of the variation called, in line with the approach used by the 1,000 genome project, we then checked, with the same 10bp window margin, if it was present within the October 2010 release of 1000 Genome only, and in this case classified as “described only in 1000 Genome”, otherwise it was classified as “novel”.

In the annotation as “described” or “novel”, when multiple INDELs have been reported within the 10bp window in ENSEMBL or 1000 Genome, we gave priority to the one with same length and sequence, then to the position, in order to choose the closest if a variant of the same length was not available.

The decision making algorithm used in our annotation script is represented in Figure S1.

# Sample variability

When inspecting the variability of the proportions of different consequences across the samples (see Figure S3), we observed that the variants already described in ENSEMBL are quite uniform within the different individuals. Novel INDELs on the opposite show a greater variability.

This may be due to the number of alleles sometimes present, each one with different consequence, but it may also suggest a role for INDELs (mostly rare, therefore accounting for private variation) in the genetics of disease. No specific statistics has been performed at this stage, but such a qualitative observation highlights the need for a deeper investigation on this issue.
